# Removal of an Encysted Fibrous Polypus, 4½ Inches in Diameter, from the Uterus, with the “Ecraseur”

**Published:** 1863-09

**Authors:** Morton Monroe Eaton

**Affiliations:** Peoria, Illinois


### REMOVAL OF AN ENCYSTED FIBROUS POLYPUS, 4½ INCHES IN DIAMETER, FROM THE UTERUS, WITH THE "ECRASEUR."

By MORTON MONROE EATON, M. D.,

*Of Peoria, Illinois,*

Mrs. W., of this city, consulted me about 3 years since, relative to her irregular menstruation, and great pain in the womb. On a digital examination, I perceived that the uterus was enlarged to the size of a fecundated womb, 3 months along—by the speculum I found the cervix uteri healthy, the os closed, &c. She gave the history of her case as follows: "She had not been well for 8 years, when her last child was born. Her menstrua had been too abundant, recurring every 2 or 3 weeks. Her appetite was, and had been, good, though she was weakly from the great loss of blood. She had consulted and been treated by several good physicians, each of whom gave a different diagnosis, and pursued a variety of methods for her cure, all equally without giving more than a temporary relief, all finally saying they were at a loss as to what was the matter and prognosed that she never could be cured.

I told her that she had something in her womb, whether a fœtus or a tumor I could not tell; though from what she said I thought it was a tumor of some kind. I gave her Ferri Pulv. and Strychnia as a tonic, under the use of which she rapidly improved every way--when I lost sight of her till about May 25th, '63, when I was called to her. Found her suffering something like labor pains. A digital examination found the os uteri dilated to the size of a half dollar, through which I could detect a globular body, apparently as large as a child's head, in the womb, very hard and unyielding. I was now sure I had a fibrous tumor in the womb, as she evidently was not pregnant, for the abdomen was not much enlarged. She was flowing freely at this time. I gave ergot tea, and watched progress. The tumor came down through the os, completely filling the pelvis, so I could not pass my finger up on either side of it, and coming within an inch of the external genitalia. At this time I was called to Vicksburg, Miss., to attend to our wounded, and left the case with Dr. Dickinson who diagnosed a fibrous tumor, but as the hæmorrhage had ceased, gave her only anodynes and tonics a few days when she was able to be around.

July 1st, soon after my return from the South, I was called to the case again, to find her flowing profusely and the tumor "in statu quo." I recommended its removal, and was seconded by Drs. Frye, Dickinson, Hamilton and Case, whom I invited to see the case, but the patient would not consent to the operation, saying, she would die anyhow; so I only gave anodynes and astringents, with tonics. I told her she need never send for me again unless she wished me to remove the tumor, as it was useless, the hæmorrhage was sure to recur so long as the tumor remained.

August 17th, I was sent for to remove the tumor, the messenger stating she was now worse reduced than ever. I called at her residence and found her almost pulseless, with a cold sweat on all parts of her body. I gave tonics, stimulants and astringents, and applied cold to the genitals, and over the gastric region, and told them she was too weak to have the operation performed then. Under this treatment she revived, the hæmorrhage ceasing entirely after two or three days, and on August 24th, she having become much recruited and desiring me to perform the operation, (though she was still unable to sit up,) I proceeded to do so in the presence of Drs. Dickinson and Hamilton. Dr. H. administered chloroform; I lengthened the chain of the ecraseur with a wire, to get it over the tumor, after which I took up the chain 16 links, and gradually turned the thumb-screw till the chain cut through which took about ten minutes, at the slow rate, adopted to avoid all hæmorrhage after the operation. As the chain cut through, a amount of a creamy fluid escaped from the vagina, and the tumor collapsed to about one-fourth its former size when it was easily removed with the fingers. This was found (by making a section of it) to consist of yellow, elastic fibrous tissue, and to measure 2½ inches in diameter and about 7½ inches in circumference. The fluid that escaped seemed to have been infiltrated all through this elastic tissue, as there were several sinuses coming through it, in a collapsed state. No hæmorrhage followed the operation. She recovered from the chloroform well, and has had no unpleasant symptoms since. Now, a week after the operation, she is able to be around the house, and is feeling quite strong, and more comfortable than she has for 8 or 10 years.

This is the first time I have had occasion to use the ecraseur and I desire to recommend it to the profession as just the instrument for such a case. The pedicle of this tumor was an inch and a half in diameter, of solid fibrous tissue, and the removal of it with the ligature would have been both tedious and painful. With the ecraseur my patient experienced no pain or inconvenience. I believe this instrument can be advantageously used more frequently than it has been. I have recently been consulted about the removal of an ovarian tumor, in which case I think the ecraseur may be used in severing its attachments. True these large fibrous polypi are not very numerous, still, if a man only operates once in a lifetime it will pay to use the ecraseur. I intended to mention the replacement of a chronically inverted and prolapsed uterus which I have just treated, but will defer it till another time. Also a hypertrophy of the clitoris in another patient, of which I will also defer the history.

